# Proteomics Investigation of the Impact of the *Enterococcus faecalis* Secretome on MCF-7 Tumor Cells

**DOI:** 10.3390/ijms241914937

**Published:** 2023-10-05

**Authors:** Moudi A. Alwehaibi, Mysoon M. Al-Ansari, Assim A. Alfadda, Reem Al-Malki, Afshan Masood, Anas M. Abdel Rahman, Hicham Benabdelkamel

**Affiliations:** 1 Department of Botany and Microbiology, College of Science, King Saud University, Riyadh 11451, Saudi Arabia; modhialwehaibi@gmail.com (M.A.A.); reem-hm876@hotmail.com (R.A.-M.); 2 Proteomics Resource Unit, Obesity Research Center, College of Medicine, King Saud University, Riyadh 11461, Saudi Arabia; aalfadda@ksu.edu.sa (A.A.A.); afsmasood@ksu.edu.sa (A.M.); 3 Department of Medicine, College of Medicine and King Saud Medical City, King Saud University, Riyadh 11451, Saudi Arabia; 4 Metabolomics Section, Department of Clinical Genomics, Center for Genome Medicine, King Faisal Specialist Hospital and Research Centre (KFSHRC), Riyadh 11211, Saudi Arabia; aabdelrahman46@kfshrc.edu.sa; 5 Department of Biochemistry and Molecular Medicine, College of Medicine, Alfaisal University, Riyadh 11533, Saudi Arabia

**Keywords:** MCF-7 cell line, microbiota, proteomic profile, breast cancer, *Enterococcus faecalis*

## Abstract

Breast cancer is the most prevalent form of cancer among women. The microenvironment of a cancer tumor is surrounded by various cells, including the microbiota. An imbalance between microbes and their host may contribute to the development and spread of breast cancer. Therefore, the objective of this study is to investigate the influence of *Enterococcus faecalis* on a breast cancer cell line (MCF-7) to mimic the luminal A subtype of breast cancer, using an untargeted proteomics approach to analyze the proteomic profiles of breast cancer cells after their treatment with *E. faecalis* in order to understand the microbiome and its role in the development of cancer. The breast cancer cell line MCF-7 was cultured and then treated with a 10% bacterial supernatant at two time points (24 h and 48 h) at 37 °C in a humidified incubator with 5% CO_2_. Proteins were then extracted and separated using two-dimensional difference (2D-DIGE) gel electrophoresis, and the statistically significant proteins (*p*-value < 0.05, fold change > 1.5) were identified via matrix-assisted laser desorption/ionization–time-of-flight mass spectrometry (MALDI-TOF-MS). The protein fingerprints showed a differential protein expression pattern in the cells treated with *E. faecalis* for 24 and 48 h compared with the control. We found 58 statistically significant proteins changes in the MCF-7 breast cancer cells affected by *E. faecalis*. Kilin and transgelin were upregulated after 24 h of treatment and could be used as diagnostic and prognostic markers for breast cancer. In addition, another protein involved in the inhibition of cell proliferation was coiled-coil domain-containing protein 154. The protein markers identified in this study may serve as possible biomarkers for breast cancer progression. This promotes their future uses as important therapeutic goals in the treatment and diagnosis of cancer and increases our understanding of the breast microbiome and its role in the development of cancer.

## 1. Introduction

Cancer is a major health concern and the second leading cause of death worldwide; it is a multifactorial disease caused by genetics or environmental factors that lead to uncontrollable cell division. Cancer affects multiple organs and tissues such as the liver, lung and breast [1]. Breast cancer (BC) is the most frequent cancer among females worldwide, with a high mortality rate [2]. Nearly one in eight women are diagnosed with breast cancer in their lifetime, as reported by the American Cancer Society [3]. Breast cancer’s biological and clinical behavior varies from patient to patient, making it difficult to predict disease evolution and patient outcomes. Therefore, biomarkers are required to assist clinicians in selecting the best treatment for each patient. Various studies on breast cancer have also determined that almost all cancers release their components into circulation. Cancer cells can release microvesicles called extracellular vesicles (EVs) or exosomes into the extracellular environment. These vesicles play an important role in cell-to-cell communication by transferring proteins, nucleic acids, and lipids from one cell to another [4].

The tumor microenvironment (TME) comprises various components, including cancer cells and host stromal cells, all of which are embedded in the extracellular matrix. The TME plays an influential role in tumor progression and invasion by affecting the biological behaviors of cancer cells, such as cell-to-cell communication and evading the immune response [1,5,6,7,8,9]. The microenvironment also contains a variety of microbiota. Microbiota are defined as microorganisms that live symbiotically in different regions of the body and play significant roles in human health. Dysbiosis of microbiota may lead to systemic disease and inflammation. Changes in a distinct microbiome may contribute to the development of cancer and affect tumor growth [10,11,12,13,14]. Breast tumors have demonstrated the presence of unique bacterial populations compared to the microbiome surrounding normal mammary gland tissue. Several studies have found a greater degree of bacterial colonization in women with breast cancer, including *Bacillus*, *Enterobacteriaceae*, *Staphylococcus*, *Fusobacterium*, *Atopobium*, *Gluconacetobacter*, *Hydrogenophaga* and *Lactobacillus* [6,12,13].

*Enterococcus faecalis* is one of the most common *Enterococcaceae* species found in the human gut and urogenital tract. Conversely, a few studies found that the metabolites produced by *E. faecalis* have an apoptotic effect against different cancer cell lines and could be used as alternative pharmaceutical substances. The functional properties of potent anticancer chemicals (protein compounds) and the mechanisms through which *E. faecalis* promotes the initiation and progression of cancer remain unknown. They should be properly researched [15,16].

A proteomics analysis is the study of proteins and represents one of the most important disciplines that provides high-throughput insight into the mechanisms of biological processes. Mass spectrometry (MS)-based proteomics technology is currently used for the identification of disease-specific biomarkers by profiling protein signatures over a wide dynamic range [17]. Differential analyses of cancerous and non-cancerous breast cell line proteomes may provide valuable proof of new proteins related to the progression of breast cancer. They also contribute to a better understanding of microbes and their molecular effects. Ultimately, the investigation of cancer and the outcomes of bacterial biomarkers may assist in disease diagnosis and could be used in the future as promising therapeutic targets [18,19,20,21]. A recent study hypothesized that the normal microbiome of the breast influences breast cancer metabolism. The study tested the *Escherichia coli* secretome to profile the most significant bacterial metabolites in the MDA-MB-231 breast cancer cell line, using liquid chromatography–mass spectrometry. The findings were the first to reveal that the *E. coli* secretome modulates the energy metabolism of BC cells and that the secretion of metabolic molecules could alter the metabolism of BC cells to maintain their survival [22].

Using 2D-DIGE and MALDI-TOF-MS, this study focused on proteomics changes in the breast cancer cell line MCF-7 which were influenced by the signaling crosstalk of *Enterococcus faecalis*. In addition, the identification of biomarkers of the *Enterococcus faecalis* secretome may provide a new line of investigation for gaining further insights into disease diagnosis, and these biomarkers could be used in the future as promising therapeutic targets that are significant in the treatment and diagnosis of cancer, using *Enterococcus faecalis* as a conventional method for cancer therapy.

## 2. Results

### 2.1. E. faecalis Supernatant Effects the Expression of MCF-7 Cell Line Proteins 

**Figure 1:**
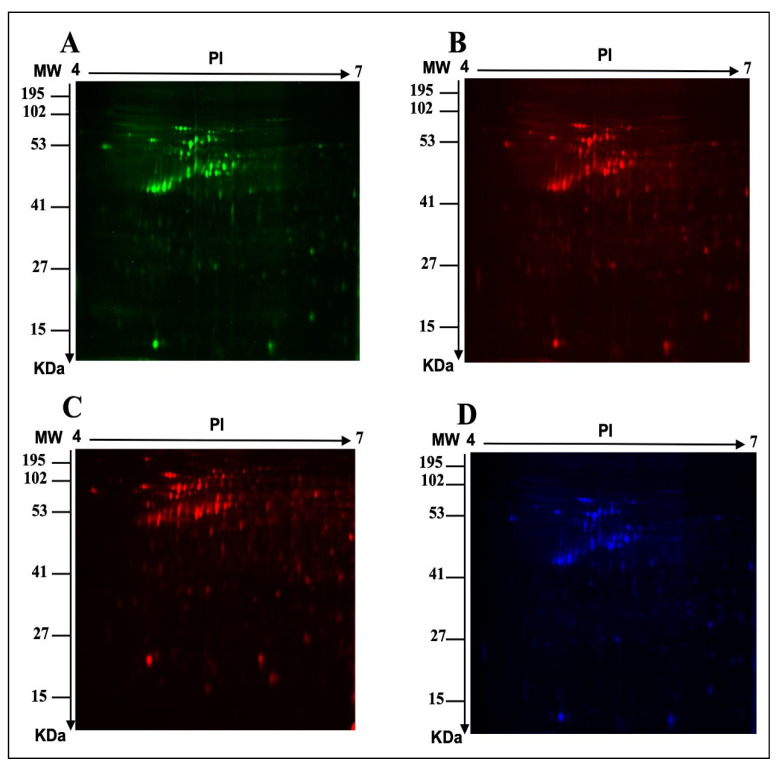
Representative fluorescent proteins of two-dimensional difference in gel electrophoresis (2D-DIGE). (**A**) Control, consisting of non-treated MCF-7 cells labeled with Cy3, (**B**) MCF-7 cells treated with *E. faecalis* for 24 h and labeled with Cy5, (**C**) MCF-7 cells treated for 48 h and labeled with Cy5, and (**D**) pooled internal control labeled with Cy2.

**Figure 2:**
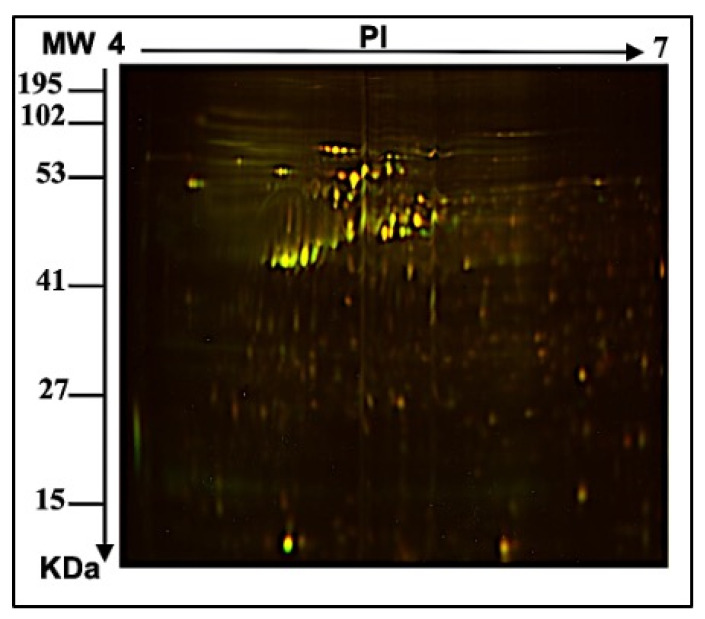
Merged 2D-DIGE gels of all samples. Fluorescent protein demonstrative of the two-dimensional difference in gel electrophoresis (2D-DIGE) in merged images labeled with Cy3, Cy5, and Cy2.

**Figure 3:**
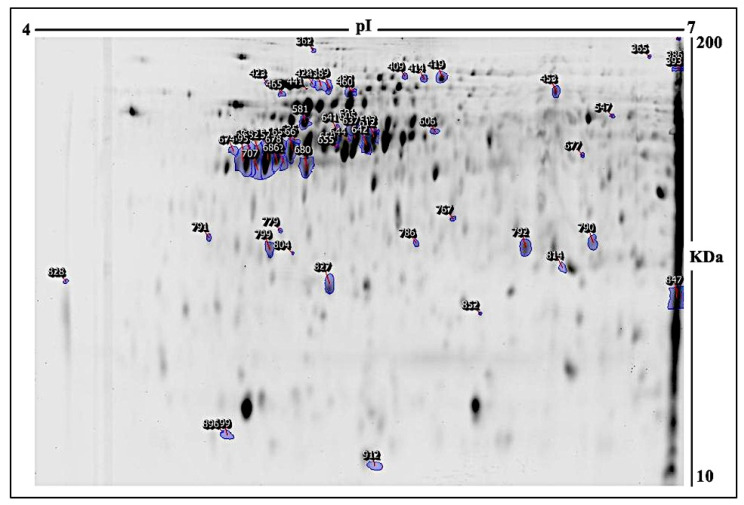
Indicated protein spots. A representative 2D-DIGE gel with numbered spots indicating differentially abundant proteins (defined as a fold change ≥ 1.5, *p* ≤ 0.05) treated with *E. faecalis* for 24 or 48 h compared to the control, identified via matrix-assisted laser desorption/ionization–time-of-flight mass spectrometry (MALDI-TOF-MS). MW—protein molecular weight; pI—isoelectric point.

To study the role of bacterial microbiota in the protein profile of MCF-7 cells, an *E. faecalis* supernatant was used as a part of the breast microbiome to test the indirect effect on the MCF-7 breast cell line. To perform a proteomic analysis using 2D-DIGE and MALDI-TOF-MS for fluorescent protein profiling, the samples were labeled with Cy3 (Ctrl), treated with *E. faecalis* for 24 h and 48 h, and labeled with Cy5, and the pooled internal control was labeled with Cy2 (Figure 1A, Figure 1B, Figure 1C, Figure 1D respectively). Figure 2 shows the merged 2D-DIGE gels of the 24 h, 48 h, and Ctrl samples. Therefore, using an ANOVA (*p* ≤ 0.05; fold-change ≥ 1.5), Figure 3 shows that the expression of 58 out of 66 proteins was significantly dysregulated in the 24 h and 48 h treated cells when compared to the control. Across five proteomic gels, the normalization and quantitative differential analysis of protein levels were attained using the internal standard Cy2 labeling.

Therefore, PMFs successfully identified 66 of the total protein spots excised from the preparative gel via MALDI-TOF mass spectrometry, which expressed 58 spots as unique protein sequences which were matched to entries in the SWISS-PROT database with high confidence scores via Mascot (Table 1). the sequence coverage of the proteins identified via PMFs ranged from 11% to 83%. Figure 3 shows that some variants of the same protein were found at different locations in the preparative gel. Table 1 shows that among the 24 h treated samples, 33 protein spots were significantly upregulated, 12 were downregulated, and 13 were non-significant when compared to the experimental control. Moreover, 16 protein spots were significantly upregulated, 15 were downregulated, and 27 were non-significant in the samples treated for 48 h compared to the control subjects. Among the 33 functional proteins upregulated after 24 h, nitric oxide synthase (upregulated 1.5-fold; *p* = 0.003) and transgelin (upregulated 1.5-fol;, *p* = 0.004) may have important roles in the progression of BC. In addition, within 48 h, the dysregulation of several proteins such as ATP-dependent RNA helicase DDX3X (downregulated −1.5-fold; *p* = 0.06), ATP synthase subunit alpha mitochondrial (downregulated −1.5-fold; *p* = 0.06), and ATP synthase subunit beta mitochondrial (up 1.5-fold; *p* = 0.02) may play roles as biomarkers of cancer progression and metastasis. Among the identified proteins, Actin, cytoplasmic 2, 60 kDa heat shock protein, mitochondrial, and AN1-type zinc finger protein 3 were found in more than one spot on the gels, which could be associated with post-translational modifications, cleavage via enzymes, or the presence of different protein species. Thus, the MCF-7 cell line was indirectly affected by the *E. faecalis* supernatant, as shown via the dysregulation of protein expression.

### 2.2. Principal Component, Cluster Analysis, and Heatmap of Significant Proteins

**Figure 4:**
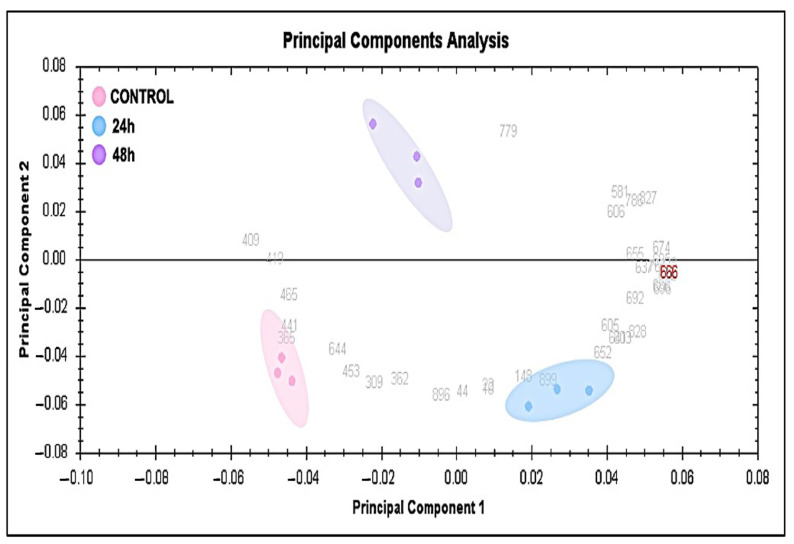
Principal component analysis of the proteomic dataset. Pink dots represent control samples, blue dots represent treated MCF-7 cells after 24 h, and purple dots indicate treated MCF-7 cells after 48 h. This explained 50.30% of the selected spot variability values. Colored dots and numbers indicate the gels and spots, respectively.

**Figure 5:**
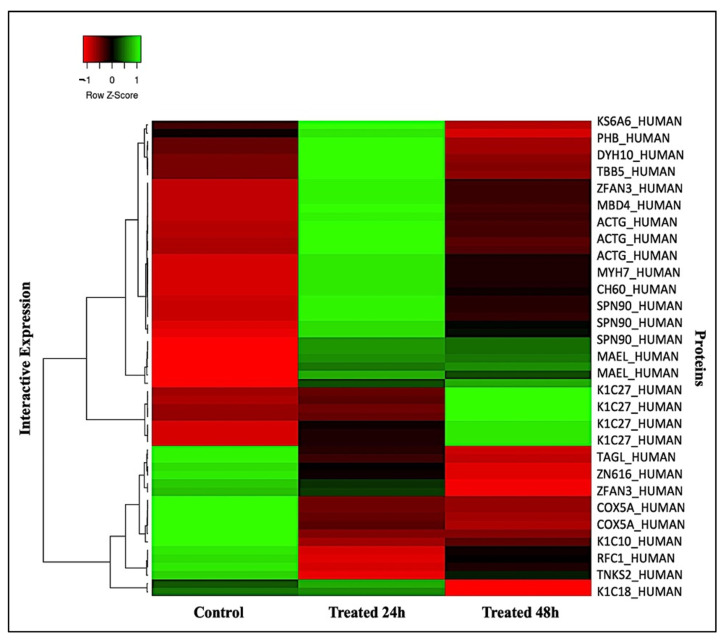
Heatmap representation of the differentially expressed protein spots. Interactive protein expression in treated MCF-7 cells (24 h and 48 h) and their relative control.

Subsequently, a multivariate analysis of the protein abundance data was performed using the Progenesis Same Spots software. The gel images were grouped such that nine samples from the control and the groups treated for 24 h and for 48 h formed three groups. The data were filtered so that only the 58 spot features exhibiting statistically significant (ANOVA, *p* ≤ 0.05) changes in abundance which were present on all gel images and identified via MS were considered. Figure 4 shows that the samples were colored according to their group, and score plots were obtained for all three study groups, taking 50.30% as the cut-off score. A principal component analysis (PCA) revealed that the three groups clustered distinctly from one another based on different time points. The PCA model demonstrated that the Ctrl group, the group treated for 24 h, and the group treated for 48 h clustered in a two-dimensional score plot, indicating that the proteomics profiles were significantly different between these three groups. Simultaneously, a hierarchical clustering analysis of abundant spots showed clusters of different expression patterns (Figure S1A,C). The clustering pattern demonstrates that the changes in the protein intensity of selected spots treated for 24 h and 48 h compared to the control samples were significantly different (Figure S1B,D). Moreover, Figure 5 displays all the significant proteins identified via MALDI- TOF-MS which were used to create a heatmap using the average of normalized values. Shades of red indicate high levels of expression and shades of green indicate low levels of expression. When comparing the experimental control to the samples treated for 24 h and 48 h, the heatmap revealed that the most recognized proteins had upregulated expression patterns. Therefore, the proteomic profiles of the significant proteins were significantly different among the three groups.

### 2.3. Bioinformatics Analysis

#### 2.3.1. Interactions of Identified Proteins and Network Connectivity Mapping Using Ingenuity Pathway Analysis (IPA)

**Figure 6:**
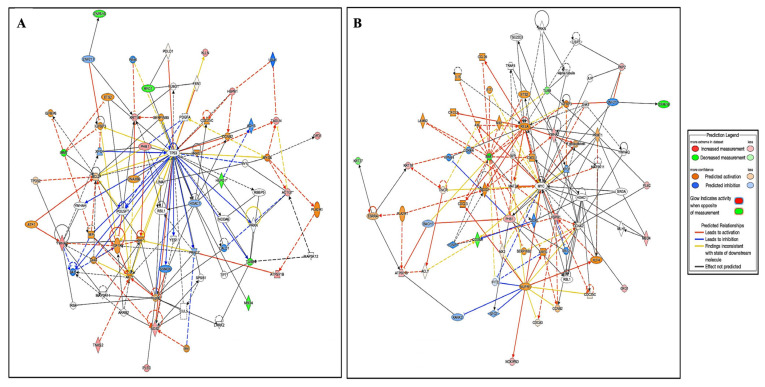
Protein interaction analysis and biological pathways related to significantly identified proteins. The most enriched interaction network of differentially expressed proteins between different groups. (**A**) Interaction network of differentially expressed proteins in cells treated with *E. faecalis* for 24 h compared with the control. The central nodes of the pathway related to TP53, HDAC 1, and BAD signaling were found to be dysregulated between the two states. The highest interaction network pathway (score = 37). Red nodes indicate upregulated proteins; green nodes indicate downregulated proteins; uncolored nodes were proposed via an IPA. Solid lines indicate direct molecular interactions, and dashed lines represent indirect interactions. (**B**) Interaction network of differentially expressed proteins in cells treated with *E. faecalis* for 48 h compared to the control. The central nodes of the pathway related to HSPB1, RELA, and P glycoprotein signaling were found to be upregulated between the two states. The highest interaction network pathway (score = 34).

**Figure 7:**
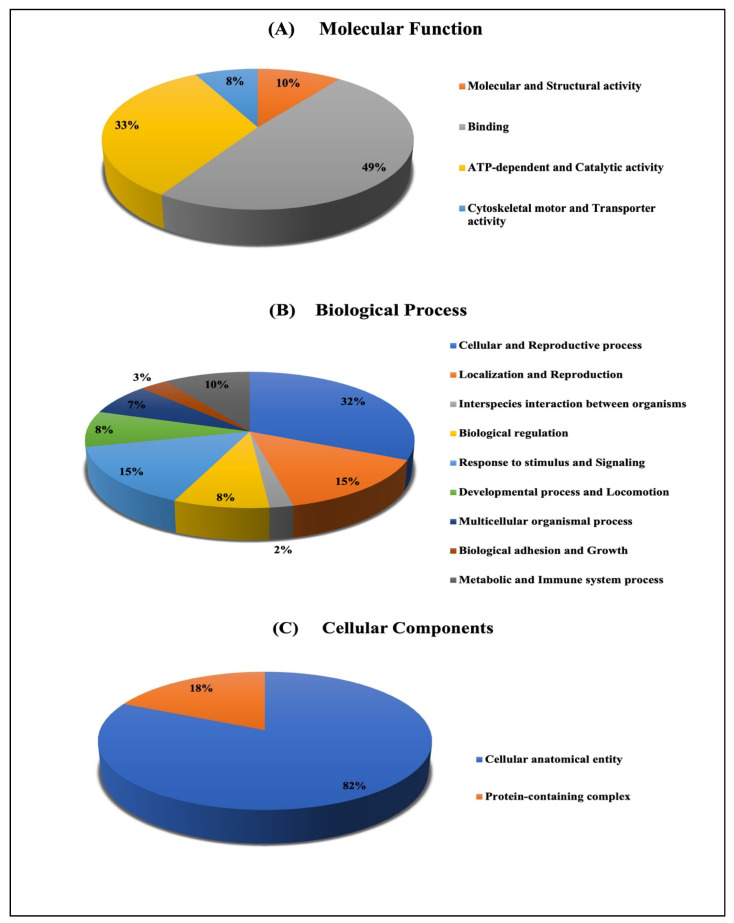
Comparative depiction of the differentially abundant proteins, categorized into groups according to their molecular function (**A**), biological process (**B**), and (**C**) proteins, according to their cellular components in the groups treated for 24 h and 48 h compared to the control.

A bioinformatics analysis using IPA was performed for all 58 proteins that were differentially dysregulated between the cells treated with *E. faecalis* for 24 h and 48 h compared to the Ctrl. The analysis revealed that the proteins interacted directly or indirectly via protein networks following treatment with *E. faecalis* for 24 h/control (Figure 6A) and 69 proteins following treatment with *E. faecalis* for 48 h/control (Figure 6B). To construct a protein–protein interaction network, the software computes a score based on the best fit from the input dataset and the biological functions database. The resulting network is enriched for proteins with interactions in which the interacting proteins are represented by nodes and a line denotes their biological relationships. Protein interaction networks were identified for the proteins exhibiting differential expression profiles in the cells treated with *E. faecalis* for 24 h/control. The proposed highest interaction network pathway (score = 37) was related to cancer, organismal injury and abnormalities, and reproductive system disease (Figure 6A). As shown in Figure S2A, the canonical pathways were sorted by decreasing (*p*-value) enrichment. Death Receptor Signaling (*p*-value: 4.33 × 10^−4^; overlap: 3.2%), the MSP-RON signaling pathway (*p*-value: 3.67 × 10^−3^; overlap: 3.4%), ILK signaling (*p*-value: 3.58 × 10^−3^; overlap: 1.5%), the Glycerol-3-phosphate shuttle (*p*-value: 3.15 × 10^−3^; overlap: 50%), and Base Excision Repair (BER) (*p*-value: 2.18 × 10^−3^; overlap: 4.5%) pathway the five most interesting enriched canonical pathways. Moreover, In Figure 6B, 69 interaction networks were identified for proteins exhibiting differential expression profiles. The network pathway with the highest score (score = 34) was related to cell death and survival, cellular developmental, and cellular growth and proliferation. the canonical pathways were sorted by decreasing the log (*p*-value) of enrichment, such as 14-3-3-mediated signaling (*p*-value: 6.35 × 10^−3^; overlap: 1.6%), oxidative phosphorylation (*p*-value: 4.54 × 10^−3^; overlap: 1.9%), ERK/MAPK signaling (*p*-value: 1.76 × 10^−2^; overlap: 0.9%), glucocorticoid receptor signaling (*p*-value: 1.41 × 10^−2^; overlap: 0.6%), and mitochondrial dysfunction (*p*-value: 1.07 × 10^−2^; overlap: 1.2%), as shown in Figure S2B. A network pathway analysis was conducted to gain insight into the molecular mechanisms of the differentially expressed proteins through biological function annotations, protein–protein interaction networks, and the discovery of potential biomarkers (Figure 7).

#### 2.3.2. Classification of Key Proteins Based on Function

For the classification of the identified proteins according to their molecular function (Figure 4) and biological function, the Protein Analysis Through Evolutionary Relationships (PANTHER) system was used (Figure 7). The functional categories showed most of the differentially expressed proteins among the up- and downregulated proteins between those treated with *E. faecalis* for 24 h compared to the control group and those treated with *E. faecalis* for 48 h compared to the control groups. The molecular function determination showed multiple protein activities, including molecular and structural activities, binding activity, ATP-dependent activity, catalytic activity, cytoskeletal activity, and transporter activity (Figure 7A). Additionally, most of the identified proteins were involved in biological processes such as cellular and reproductive processes, localization, and reproduction, interspecies interactions between organisms, biological regulation and adhesion, response to stimulus, and signaling. This was followed by developmental processes, locomotion, multicellular organismal processes, biological adhesion and growth, and metabolic and immune system processes (Figure 7B). In terms of biological activities, most of the proteins were found to be involved in cellular components, for instance, the cellular anatomical entity and protein-containing complex (Figure 7C).

## 3. Materials and Methods

### 3.1. Cell and Bacterial Culture

#### 3.1.1. Cells and Cell Culture

The cell line was obtained from the King Faisal Specialist Hospital & Research Centre. The epithelial breast cancer cell line (MCF-7) is characterized as estrogen/progesterone-positive and human epidermal growth factor receptor 2 (HER2)-negative. The cell line was cultured (1 × 10^6^ cells/mL) in plates (100 × 20 mm) using Dulbecco’s Modified Eagle’s Medium/Nutrient Mixture F-12 Ham (DMEM/F-12) as a complete medium mixed with 10% Fetal Bovine Serum (FBS), 1% L-glutamine, and 1% penicillin/streptomycin, following the manufacturer’s instructions. The cells were then incubated at 37 °C in a humidified incubator with 5% CO_2_ until they reached 80% confluency. The cells were then washed with Phosphate-Buffered Saline (PBS) and treated with an *Enterococcus faecalis* supernatant (explained below) [23,24].

#### 3.1.2. Bacterial Culture and Preparation

*Enterococcus faecalis* (ATCC 29212) were grown in a Luria–Bertani (LB) broth medium and incubated at 37 °C for 24 h. The optical density (OD) of the bacterial culture was measured using a spectrophotometer (Libra S22, Biochrom Ltd., Cambridge, UK) after 24 h at a wavelength of 600 nm. The supernatant was collected after centrifugation, filtered through a 0.2 μm filter, and stored at −80 °C for further use [25,26,27]. The collected media were evaluated using the re-culture protocol to prevent potential live bacteria in the supernatant from contaminating the cell lines.

#### 3.1.3. Indirect Bacterial Effect Using SF-CM Media on MCF-7 Cells

To investigate the indirect bacterial effect on MCF-7 cells, the cells were cultured in serum-free conditioned medium (SF-CM) (DMEM/F-12, supplemented with 0.5% FBS, 1% L-glutamine and 1% penicillin/streptomycin) for 24 h at 37 °C in 5% CO_2_. Next, 10% of the bacterial supernatant was mixed with each cell culture (1 × 10^6^), and non-treated cells were used as controls. The cells were incubated at 37 °C in 5% CO_2_ for different time periods (24 h and 48 h). Following incubation, the cells were collected using ice-cold PBS and a cell scraper (280 mm), centrifuged, and stored at −80 °C for further use, as previously described [28,29].

### 3.2. Proteomic Analysis

#### 3.2.1. Protein Extraction

To perform a proteomic analysis of our samples, 50 µg of protein was extracted as previously described [19,20]. Briefly, the cells (5 million cells) were resuspended in lysis buffer (1.5 mL of a 30 mM Tris buffer with a pH of 8.8 containing 7 M urea, 2 M thiourea, 2% 3-[(3-cholamidopropyl) dimethylammonio]-1-propanesulfonate (CHAPS), and 1× protease inhibitor mix). The samples were gently rocked (20 min; room temperature) and then sonicated (30% pulse; two intervals of 1 min each separated by a 1 min gap). Then, 50 mM of dithiothreitol (DTT) was added, and the protein extracts were centrifuged (20,000× *g*; 40 min, 4 °C). Contaminants were removed, and the supernatants were cleaned via precipitation, using a 2D clean-up kit according to the manufacturer’s protocol (GE Healthcare, Danderyd, Sweden) [17,30].

#### 3.2.2. Protein Labeling with Cyanine Dyes

The protein pellets were solubilized in a labeling buffer (7 M urea, 2 M thiourea, 30 mM Tris (hydroxymethyl) aminomethane hydrochloride (Tris–HCl), and 4% CHAPS at a pH of 8.5). Using centrifugation, the insoluble material was pelleted at 12,000× *g* at room temperature for 5 min, after which the protein concentrations were determined in triplicate using the 2D-Quana kit (GE Healthcare, Danderyd, Sweden), and the pH was adjusted to 8.5 using NaOH (100 mM). The proteins were labeled using 400 pmol of CyDye™ DIGE Fluor dyes (GE Healthcare, Danderyd, Sweden) in 1 µL of *N*,*N*-dimethylformamide (DMF) and mixed with samples containing 50 µg of protein. The samples were then incubated on ice for 30 min in the dark. The labeling reaction was terminated by adding 1 µL of 10 mM lysine. Each sample was labeled with a fluorophore, either Cy3 or Cy5. A pool containing equal amounts of protein from each sample in the experiment was labeled with Cy2 and served as an internal standard [17,30].

#### 3.2.3. Two-Dimensional (2D) Electrophoresis, Image Scanning, and Preparative Gel

For the first dimension of separation, nine dry strips of Immobiline strips (measuring 24 cm and at a pH of 3–11; GE Healthcare, Danderyd, Sweden) were passively rehydrated (30 V; 12 h), followed by isoelectric focusing using an Ettan IPGphor IEF unit (GE Healthcare, Danderyd, Sweden). The focusing was performed at 20 °C, with 50 µA per strip. After the first dimension, the strips were equilibrated and separated on 12.5% SDS-PAGE gels using an Ettan Dalt Six device (GE Healthcare, Danderyd, Sweden). The gels were then scanned using a Sapphire Biomolecular Imager (Azure Bio systems, Dublin, OH, USA), and digitalization was performed using the image analysis software Sapphire Capture system (Azure Biosystems, Dublin, OH, USA). Preparative gels were prepared using the total protein (1 mg) obtained from a pool of equal protein amounts. The gels were then stained using Colloidal Coomassie Blue, as described previously [17,30,31].

#### 3.2.4. Statistical Analysis

Progenesis SameSpots software (version 3.3, Nonlinear Dynamics Ltd., Newcastle, UK) was used to analyze the 2D-DIGE gel images via an automated spot-detection method. The analysis included a comparison of the samples. Moreover, to detect all spots across all gels, an automatic analysis was performed, and each selected spot was verified and manually edited wherever necessary. Normalized volumes were used to identify differentially expressed spots. Statistical significance was set at a cut-off ratio of ≥1.5-fold. Each experiment was performed in triplicate.

#### 3.2.5. Protein Identification via Matrix-Assisted Laser Desorption/Ionization–Time-of-Flight Mass Spectrometry (MALDI-TOF-MS)

The Coomassie-Blue-stained gel spots were excised manually, washed, and digested according to a previously described protocol [17,30]. Finally, 0.8 μL of a mixture of tryptic peptides derived from each protein was spotted onto a MALDI target (384 MTP Anchorchip; 800 μm Anchorchip; Bruker Daltonics, Bremen, Germany). Spectra were acquired using a MALDI-TOF MS (UltraFlexTrem, Bruker Daltonics, Germany) device in the positive mode (target voltage—25 kV; pulsed ion extraction voltage—20 kV). The reflector voltage was set to 21 kV, and the detector voltage was set to 17 kV. The peptide mass fingerprints (PMFs) were calibrated against a standard mixture by assigning appropriate mono-isotopic masses to the peaks as follows: bradykinin (1–7), *m*/*z* 757.399; angiotensin I, *m*/*z* 1296.685; angiotensin II, *m*/*z* 1046.54; rennin-substrate, *m*/*z* 1758.93; ACTH clip (1–17), *m*/*z* 2093.086; and somatostatin, *m*/*z* 3147.471 (peptide calibration standard II, Bruker Daltonics, Germany). The MS spectra were recorded automatically across the mass range of *m*/*z* 700–3000, and the spectra were typically the sum of 400 laser shots. The PMFs were processed using Flex AnalysisTM software (version 2.4, Bruker Daltonics, Germany), and sophisticated numerical annotation procedure (SNAP) algorithms were used for peak detection (S/N—3; maximum number of peaks— 100; quality factor threshold—30). The MS data were interpreted using BioTools v3.2 (Bruker Daltonics, Germany), together with the Mascot search algorithm (version 2.0.04 updated 09/05/2020; Matrix Science Ltd., London, UK). The Mascot parameters were as follows: fixed cysteine modification with propionamide, variable modification due to methionine oxidation, one missed cleavage site (i.e., in the case of incomplete trypsin hydrolysis), and a mass tolerance of 100 ppm. Identified proteins were accepted as correct if they showed a Mascot score greater than 56 and *p* < 0.05, sequence coverage of at least 20%, and a minimum of four matched peptides. Not all spots of interest could be identified because some proteins were low in abundance and did not yield sufficiently intense mass fingerprints, whereas others were mixtures of multiple proteins [30,32,33].

#### 3.2.6. Principal Component Analysis, Cluster Analysis, and Heatmap

Progenesis SameSpots software (version 3.3, Nonlinear Dynamics, Newcastle upon Tyne, UK) was used to analyze the principal components at (*p* ≤ 0.05 by ANOVA). The software was also used to study the different hierarchical clustering and expression profiles of the samples. A heatmap was created by taking the average of the values normalized to each spot of significant proteins. The expression-based heat map tool is available on a web server (http://heatmapper.ca/, accessed on 15 May 2023).

#### 3.2.7. Bioinformatics Analysis

Analyses of protein interaction networks and the functions of the identified proteins were performed using Ingenuity pathway analysis (IPA) version 9.0 (Ingenuity Systems, Redwood City, CA, USA). The IPA software maps Uniport IDs into the ingenuity knowledge base, which is the largest manually curated resource combining information from all published scientific studies. This software assists in determining functions and pathways that are strongly correlated with the MS-generated protein list by overlaying the experimental expression data onto networks constructed from published interactions. Additionally, the identified proteins were classified into different categories according to their molecular function and biological function, using the PANTHER (protein analysis through evolutionary relationships) classification system (http://www.pantherdb.org, accessed on 20 May 2023) [17,30].

## 4. Discussion

*Enterococcus faecalis* is one of the most common types of *Enterococcaceae* found in the human microbiota. Several studies have shown that an imbalance in microbial collection may lead to cancer and tumor growth. According to a recent study, *E. faecalis* has the potential to suppress proliferation and induce the apoptosis of MCF-7 cells in vitro and can be used as a target for the prevention and treatment of breast cancer [34]. However, the functional analysis of and mechanism through which *E. faecalis* stimulates initiation and progression remain unclear and should be investigated properly. Several proteomic studies have been conducted to identify BC biomarkers and the stages of BC progression. However, these studies were unable to clearly define the relationship between BC and the role of microbiotic proteins in the progression or regression of BC. A deeper understanding of the possible underlying molecular pathways is important for the development of reliable diagnostic and prognostic biomarkers for BC. To the best of our knowledge, this study is the first to investigate an untargeted proteomic approach using 2D-DIGE coupled with MALDI-TOF mass spectrometry and a bioinformatics analysis to study the proteomic profiles of BC after treatment with *E. faecalis*.

### 4.1. Significant Upregulated and Downregulated Proteins in the Samples Treated for 24 and 48 h 

The proteomics analysis yielded protein fingerprints displaying a pattern of differentially expressed proteins in the cells treated with *E. faecalis* for 24 h and 48 h compared to the experimental control. This study found 58 statistically significant proteins, 45 of which proteins (33 upregulated and 12 downregulated) were differentially expressed in the group treated for 24 h compared to the control group, whereas 31 proteins (16 upregulated and 15 downregulated) were differentially expressed in the group treated for 48 h compared to the control group. This illustrates the downregulation and non-significant presence of pericentrin and keratin type I cytoskeletal 27 proteins, which are responsible for cell cycle progression. Conversely, as previously demonstrated, there was an increase in 14-3-3 protein expression in the breast cancer epithelia compared to the adjacent normal epithelia. This agrees with our results, which showed elevated levels of expression in both groups compared to the control. This protein has been linked to a variety of biological processes including cell cycle progression, proliferation, and apoptosis [35].

Furthermore, in this study, several proteins were found to be upregulated in the cells treated with *E. faecalis* for 24 h compared to the control and then downregulated after treatment for 48 h, which may have been due to protein degradation. Keratin type I cytoskeletal 18 and nitric-oxide-synthase-inducible proteins were detected. Both proteins are involved in inflammation, such as interleukin-6 (IL-6) and interleukin-8 (IL-8). These inflammatory mediators have been shown to be associated with a high risk of the metastatic progression of breast cancer, as reported in [5,9,23]. Killin is a DNA-binding protein which is involved in cell-cycle arrest and is regulated via p53 oncogenes. A previous study suggested that the killin gene could be used as a diagnostic marker for patients with an increased lifetime risk of developing breast and thyroid cancers [36]. Likewise, transgelin is an actin-binding protein that alters the shape and structure of the cytoskeleton. Hence, the loss of transgelin gene expression might be an essential step in the development of tumors and a diagnostic indicator for breast and colon cancer [37,38]. Moreover, poly [ADP-ribose] polymerase tankyrase-2 (TNKS-2) belongs to a large family of structurally and functionally diverse proteins. Wnt signaling and apoptosis are two of the many biological activities regulated by TNKS-2. TNKS proteins are recognized by RNF146, which mediates subsequent protein degradation [39]. In addition to another protein called small heat shock protein (HSPB1), which functions as a molecular chaperone, it probably maintains denatured proteins in a folding-competent state.

Another tumor suppressor protein, coiled-coil domain-containing protein 154 (CCDC154), was found to be upregulated after 24 h of treatment with *E. faecalis.* The overexpression of CCDC154 significantly inhibited cell proliferation and tumor cell growth. Instead of enhancing apoptosis, CCDC154 can suppress cell growth by inducing G2/M arrest. The G_2_/M cell cycle arrest is a checkpoint responsible for preventing any cells with damaged DNA from lasting. These data suggest that CCDC154 is a novel cell-cycle modulator as well as a potential tumor suppressor [40]. Furthermore, the ATP-dependent RNA helicase DDX3X is a member of the DEAD-box family of RNA helicases. Several studies have shown that DDX3X is a biomarker for good prognosis in cancers. In our study, it appeared poorly in BC after 48 h of treatment with *E. faecalis*, and the aberrant expression of DDX3X has poor clinical outcomes in different cancers, including breast cancer. DDX3X is also known to be involved in modulating tumor proliferation, migration, invasion, and drug resistance in many types of cancer [41,42].

### 4.2. Functions and Interactions of Identified Proteins

Similarly, two significant proteins that could be used as cancer biomarkers were mitochondrial ATP synthases. Similar to the results of the PANTHER analysis in this study, the expressed proteins have 33% ATP-dependent molecular function activity. ATPase is a multi-subunit enzyme complex (α, β) located in the inner mitochondrial membrane and is essential for oxidative phosphorylation under physiological conditions. According to our results, ATP subunit β was elevated in both groups when compared to the control, whereas ATP subunit α was decreased in both groups. Overall, the down- or upregulation of the ATP synthase subunit appears to be part of cancer adaptation via the desensitization of cells to permeability transition and cell death. In various cancer models, ATP synthase activity is correlated with tumor growth, proliferation, and metastatic behavior [37]. However, it may also stimulate apoptosis [43,44]. Various studies have highlighted this enzyme as a novel target that needs to be further characterized for anti-cancer strategies [43,44,45]. At the protein pathway level, this study observed that the incensement of the ATP synthase catalytic subunit plays a role in promoting oxidative phosphorylation. According to our IPA conical pathway results, oxidative phosphorylation (OXPHOS) was detected with a *p*-value of 4.54 × 10^−3^. In cancer tumors and cells, the OXPHOS pathway can be recognized as a target for the development of novel anticancer therapies. This pathway can be targeted by small-molecule inhibitors that can inhibit metabolism and induce oxidative damage and cancer cell death [43,46,47]. Interestingly, the ATP α synthase subunit can bind to the bacterial siderophore enterobactin, which is found in human microbiota and promotes the mitochondrial accumulation of enterobactin-derived iron ions. Enterobactin is almost exclusively produced by enterobacteria to scavenge iron from the environment. The scavenging role of enterobactin is expected to have a negative impact on certain cellular processes in the host given that siderophores are key virulence mediators of pathogens [48].

Through biological function annotations, protein–protein interaction networks, and the identification of novel biomarkers, an IPA was utilized for a network pathway analysis to obtain insights into the molecular processes of differentially expressed proteins. A conical pathway analysis showed that the cluster of proteins differentially expressed between treatment with *E. faecalis* for 24 h versus the Ctrl identified the dysregulation of the death receptor signaling pathway, MSP-RON signaling pathway, ILK signaling, glycerol-3-phosphate shuttle, and BER pathway. In contrast, a network analysis of the group treated for 48 h compared to the Ctrl showed the involvement of 14-3-3-mediated signaling, oxidative phosphorylation, ERK/MAPK signaling, glucocorticoid receptor signaling, and mitochondrial dysfunction. It has been reported that the over-activation of ERK/MAPK plays an essential role in cell apoptosis and invasion; however, its activation may also increase cell apoptosis or the pro-oncogenic entry of other signals in breast and pancreatic cancers. The ERK/MAPK conical pathway signaling demonstrates both oncogene and tumor suppressor effects depending on the TME [41,49,50]. Similarly, the overexpression of ILK signaling has been observed to play an important role in biological processes associated with tumorigenesis, including the cell proliferation and growth of cancer cells [51].

Treatment with *E. faecalis* could be considered in the future for the inhibition of tumor cell growth and to induce apoptosis in patients with breast cancer similar what was observed in the breast cancer cell line. Moreover, the results of this study can be used clinically as potential biomarkers for disease prognosis.

## 5. Conclusions

In conclusion, the current study provides a new understanding of the molecular function of the effects of *E. faecalis* on breast cancer. Treatment with *E. faecalis* may induce apoptosis and inhibit tumor growth in a breast cancer cell line. The panels of protein markers identified in this study could be considered potential biomarkers of interest for the diagnosis of breast cancer. Further investigations are required into their utilization as therapeutic targets and prognostic markers. In addition to the vast number of potential connections between the microbiome and various breast diseases, we also sought to better understand the microbiome in the context of the prognosis and treatment of breast disease.

## Figures and Tables

**Table 1:** Proteins identified to show changes in abundance after the treatment with *E. faecalis* for 24 h and 48 h compared to the control. Different values for the average ratio between the two states, with their corresponding levels of fold change and one-way ANOVA (*p* ≤ 0.05) using 2D-DIGE (analysis type: MALDI-TOF; database: SwissProt; taxonomy: homospines).

#	Spot No ^a^	Protein Name	MASCOT ID	Ratio 24/C ^b^	Exp ^c^	Ratio 48/C ^b^	Exp ^c^	*p* Value ^d^
1	309	Zinc finger protein 616	ZN616_HUMAN	−1.5	DOWN	−2.7	DOWN	0.003
2	641	Nitric oxide synthase, inducible	NOS2_HUMAN	1.5	UP	−1	NS	0.003
3	652	Transgelin	TAGL_HUMAN	1.5	UP	−1.1	NS	0.004
4	644	Tektin-2	TEKT2_HUMAN	−1.5	DOWN	−1.9	DOWN	0.004
5	827	Egl nine homolog 1	EGLN1_HUMAN	1.2	NS	1.2	NS	0.01
6	682	Dynein axonemal heavy chain 10	DYH10_HUMAN	1.7	UP	1.2	NS	0.01
7	828	Twinfilin-2	TWF2_HUMAN	3.2	UP	−1.1	NS	0.01
8	899	Spectrin beta chain, non-erythrocytic 5	SPTN5_HUMAN	1.2	NS	−1.2	NS	0.01
9	655	Keratin, type I cytoskeletal 18	K1C18_HUMAN	1.5	UP	1.2	NS	0.01
10	896	Cytochrome c oxidase subunit 5A, mitochondrial	COX5A_HUMAN	−1	NS	−1.9	DOWN	0.01
11	365	MICOS complex subunit MIC60	MIC60_HUMAN	−2.1	DOWN	−2.4	DOWN	0.01
12	428	Coiled-coil domain-containing protein 154	CC154_HUMAN	2	UP	1.4	NS	0.01
13	606	Plectin	PLEC1_HUMAN	1.5	UP	1.5	UP	0.02
14	686	Zinc finger protein 600	ZN600_HUMAN	1.8	UP	1.2	NS	0.02
15	692	Putative uncharacterized protein encoded by LINC02694	CO053_HUMAN	1.6	UP	1.2	NS	0.02
16	674	Centriole and centriolar satellite protein OFD1	OFD1_HUMAN	1.5	UP	1.5	UP	0.02
17	696	Protein maelstrom homolog	MAEL_HUMAN	1.6	UP	1.1	NS	0.02
18	786	Prohibitin 1	PHB_HUMAN	1.6	UP	1.6	UP	0.02
19	603	Microtubule-associated protein 6	MAP6_HUMAN	1.5	UP	−1.1	NS	0.02
20	767	Cartilage matrix protein	MATN1_HUMAN	1.5	UP	1.2	NS	0.02
21	581	ATP synthase subunit beta, mitochondrial	ATPB_HUMAN	1.5	UP	1.5	UP	0.02
22	779	Keratin, type I cytoskeletal 10	K1C10_HUMAN	1.1	NS	1.5	UP	0.03
23	465	Tubulin beta chain	TBB5_HUMAN	−1.5	DOWN	−1.5	DOWN	0.03
24	605	Poly [ADP-ribose] polymerase tankyrase-2	TNKS2_HUMAN	1.6	UP	−1	DOWN	0.03
25	409	Replication factor C subunit 1	RFC1_HUMAN	−1.5	DOWN	−1	NS	0.03
26	419	60 kDa heat shock protein, mitochondrial	CH60_HUMAN	−1.5	DOWN	−1.2	NS	0.03
27	453	Ankyrin repeat domain-containing protein 6	ANKR6_HUMAN	−1.5	DOWN	−1.9	DOWN	0.03
28	695	AN1-type zinc finger protein 3	ZFAN3_HUMAN	1.7	UP	1.2	NS	0.04
29	362	AN1-type zinc finger protein 3	ZFAN3_HUMAN	−1.1	NS	−1.7	DOWN	0.04
30	637	Actin, cytoplasmic 2	ACTG_HUMAN	1.5	UP	1.2	NS	0.04
31	791	14-3-3 protein zeta/delta	1433Z_HUMAN	1.8	UP	1.6	UP	0.04
32	678	*N*-acyl-aromatic-L-amino acid amidohydrolase (carboxylate-forming)	ACY3_HUMAN	1.7	UP	1.1	NS	0.04
33	386	Dynein axonemal heavy chain 9	DYH9_HUMAN	−1.5	DOWN	−1.7	DOWN	0.04
34	677	Forkhead-associated domain-containing protein 1	FHAD1_HUMAN	1.2	NS	2.3	UP	0.05
35	847	Keratin, type I cytoskeletal 27	K1C27_HUMAN	−1.2	NS	−1.5	DOWN	0.05
36	680	Plakophilin-2	PKP2_HUMAN	−1	NS	1.5	UP	0.05
37	601	Methyl-CpG-binding domain protein 4	MBD4_HUMAN	−1.5	DOWN	1.5	UP	0.05
38	665	Ribosomal protein S6 kinase alpha-6	KS6A6_HUMAN	1.5	UP	1.1	NS	0.05
39	642	Glycerol-3-phosphate dehydrogenase, mitochondrial	GPDM_HUMAN	1.5	UP	−1.1	NS	0.05
40	814	Heat shock protein beta-1	HSPB1_HUMAN	1.5	UP	1.8	UP	0.05
41	612	Actin, cytoplasmic 2	ACTG_HUMAN	1.5	UP	1.2	NS	0.05
42	691	Killin	KILIN_HUMAN	1.5	UP	1.1	NS	0.05
43	799	NCK-interacting protein with SH3 domain	SPN90_HUMAN	1.1	NS	1.5	UP	0.05
44	449	Protein tyrosine phosphatase domain-containing protein 1	PTPC1_HUMAN	1.5	UP	1.1	NS	0.05
45	707	Retinol dehydrogenase 13	RDH13_HUMAN	1.8	UP	1.2	NS	0.05
46	414	Pericentrin	PCNT_HUMAN	−1.5	DOWN	−1.2	NS	0.05
47	334	Sciellin	SCEL_HUMAN	−1.2	NS	−2.3	DOWN	0.05
48	423	Protein disulfide-isomerase	PDIA1_HUMAN	2.3	UP	4	UP	0.05
49	460	Myosin-7	MYH7_HUMAN	−1.6	DOWN	−1.8	DOWN	0.05
50	804	28S ribosomal protein S18c, mitochondrial	RT18C_HUMAN	2	UP	1.5	UP	0.05
51	393	ATP synthase subunit alpha, mitochondrial	ATPA_HUMAN	−1.6	DOWN	−1.5	DOWN	0.05
52	438	AN1-type zinc finger protein 3	ZFAN3_HUMAN	1.5	UP	1.2	NS	0.05
53	912	ATP-dependent RNA helicase DDX3X	DDX3X_HUMAN	1.1	NS	−1.5	DOWN	0.05
54	547	60 kDa heat shock protein, mitochondrial	CH60_HUMAN	1.1	NS	1.5	UP	0.05
55	790	Probable ATP-dependent RNA helicase DDX60	DDX60_HUMAN	1.5	UP	1.6	UP	0.05
56	792	Myosin-13	MYH13_HUMAN	1.5	UP	1.8	UP	0.05
57	487	60 kDa heat shock protein, mitochondrial	CH60_HUMAN	−1.3	DOWN	−1.3	DOWN	0.05
58	635	Myosin-4	MYH4_HUMAN	1.5	UP	1.2	NS	0.05

^a^ Protein accession number for the SWISSPROT Database. ^b^ Ratio between the groups, UP = upregulation; Down = downregulation; NS = non-significant. ^c^ Protein expression between the groups. ^d^ *p*-Value (ANOVA).

## Data Availability

Upon reasonable request, the datasets used and/or analyzed in this study are available from the corresponding author.

## Supplementary Materials

The following supporting information can be downloaded at: https://www.mdpi.com/article/10.3390/ijms241914937/s1, Figure S1: Expression profiles; Figure S2: Conical pathways; Figure S3: Study workflow.
