# Supplementary material for: Proteomics Investigation of the Impact of the Enterococcus faecalis Secretome on MCF-7 Tumor Cells
Source: Int J Mol Sci. 2023 Oct 5;24(19):14937. doi: 10.3390/ijms241914937 (PMC10573200; doi:10.3390/ijms241914937)
Supplement: Supplementary file 1 [file ijms-24-14937-s001.zip › ijms-2619164-supplementary.pdf]

Figure S1: Expression profiles were separated into clusters of expression patterns. (A) Each line represents the standardized abundance of a spot across all gels and belongs to treated for 24h compared to the control cluster generated by hierarchical cluster analysis. (B) Spots with decreased abundance indicate 12 downregulated proteins in treated MCF-7 after 24h when compared to the experimental control. Spots with decreased abundance indicated 15 downregulated proteins in treated MCF-7 cells after 48h when compared to the control. (C) Each line represents the standardized abundance of a spot across all gels and belongs treated for 48h compared to the control cluster generated by hierarchical cluster analysis, (D) Spots with increased abundance indicate the 33 proteins upregulated in treated MCF-7 after 24h compared to the experimental control. Spots with increased abundance indicated that the 16 proteins were upregulated with *E. faecalis* for 48h compared to the control (Progenesis Same Spots).

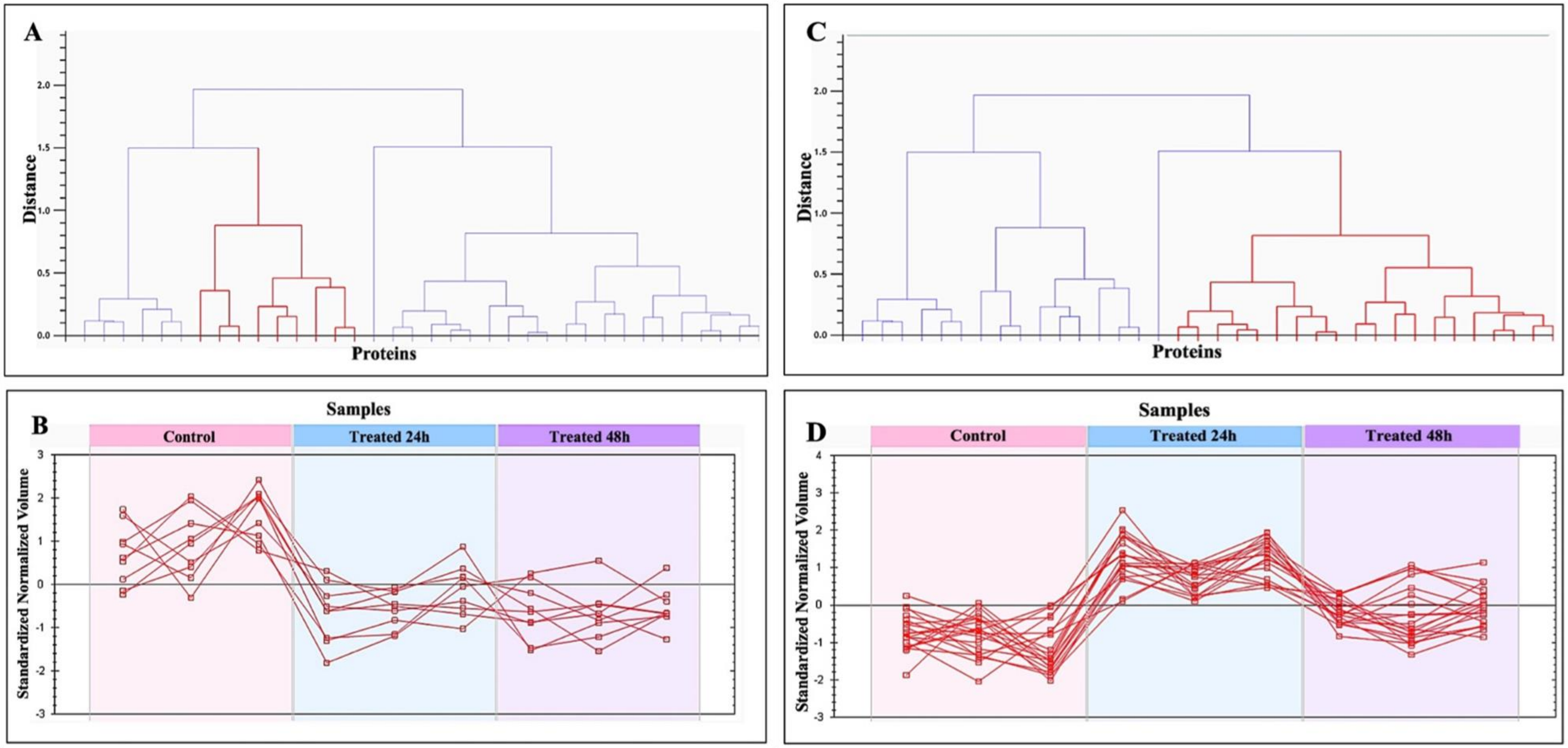

Figure S2: (A) 23 top canonical pathways identified using ingenuity pathway analysis (IPA) ranked by p-value after 24h, (B) 9 top canonical pathways identified using ingenuity pathway analysis (IPA) ranked by the P-values AFTER 48h.

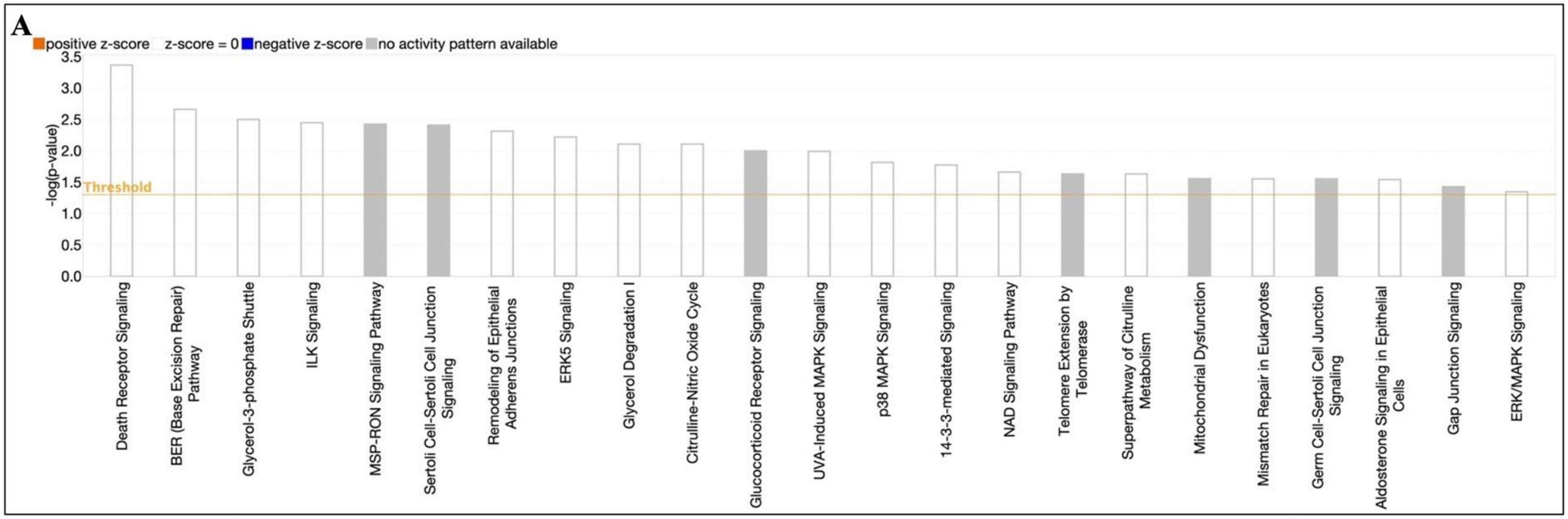

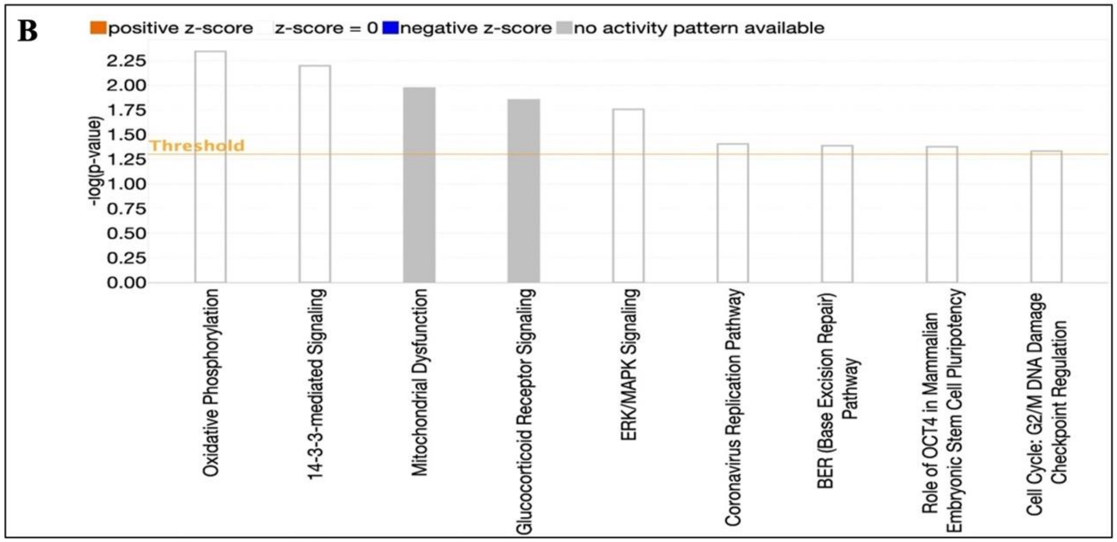

Table S1: Experimental design: 9 samples run on 5 2D-PAGE gels, samples were labeled randomly with Cy3 and Cy5, and a pooled sample was used as an internal standard and was stained with Cy2.

Gel	Cy3	Cy5	Cy2
1	N-1	T24-1	Pooled sample
2	T48-1	N-2	Pooled sample
3	N-3	T24-2	Pooled sample
4	T24-3	T48-2	Pooled sample
5	T48-3 B	-	Pooled sample
N: NON-TREATED, T24: TREATED AT 24 TIME POINT, T48: TREATED AT 48 TIME POINT.			

Figure S3: Study workflow

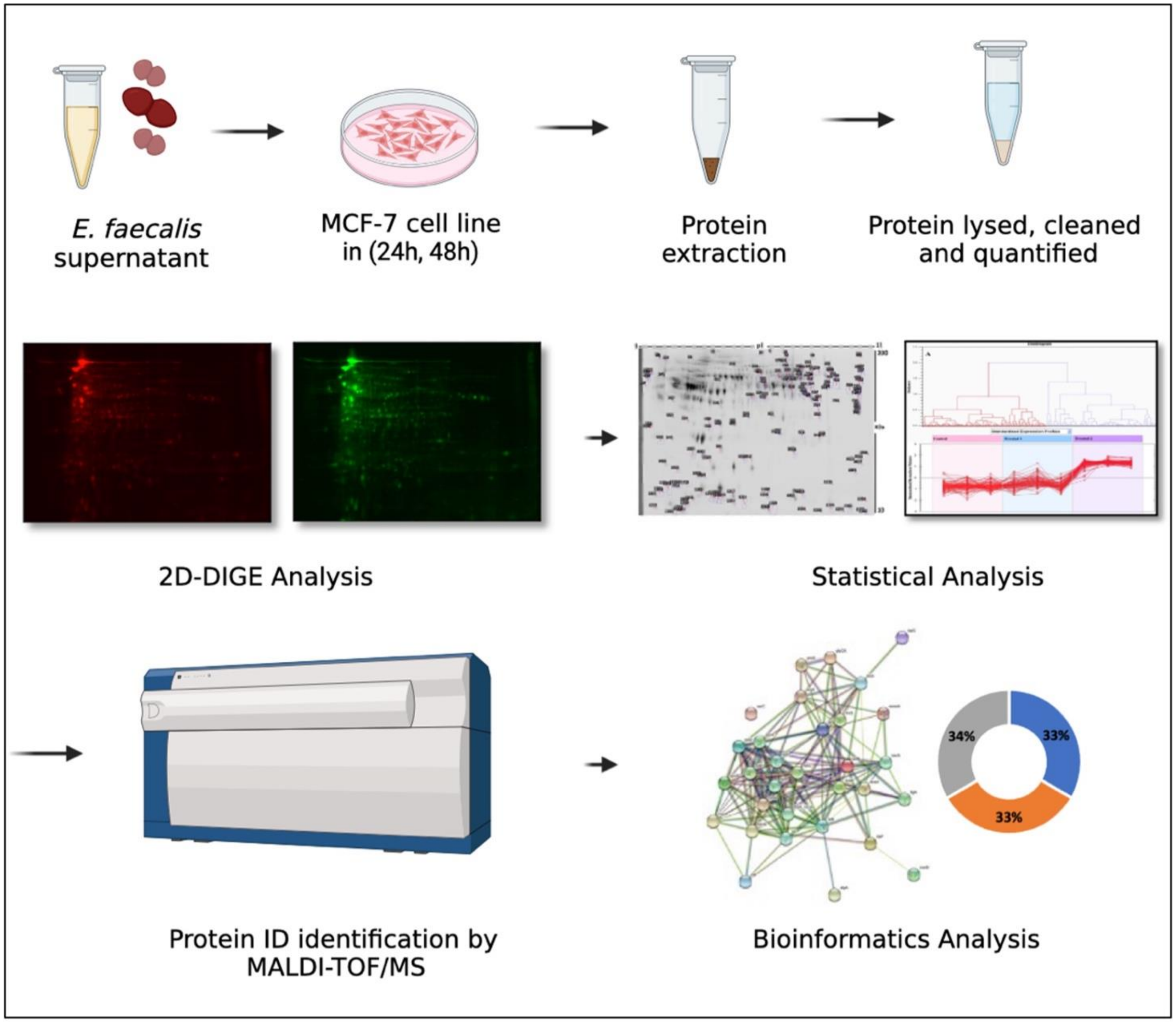

Table S2: Mass spectrometry list of significant differentially abundant proteins between liraglutide pre-treated and post –treated states identified in urine samples, using 2D-DIGE with. Protein name, accession number, Mascot score, MS % coverage, protein MW and pI values according to Uniprot database are listed. a Protein accession number for SWISSPROT Database.

Sl no:	Spot No[a]	Accession No	Protein Name	MASCOT ID	Pi[b]	MW[C]	Cov%[d]	Score[e]
1	309	Q08AN1	Zinc finger protein 616	ZN616_HUMAN	9.6	93066	22	63
2	641	P35228	Nitric oxide synthase, inducible	NOS2_HUMAN	8.20	132573	19	57
3	652	Q01995	Transgelin	TAGL_HUMAN	8.8	22653	46	65
4	644	Q9UIF3	Tektin-2	TEKT2_HUMAN	5.3	50155	30	57
5	827	Q9GZT9	Egl nine homolog 1	EGLN1_HUMAN	8.8	46847	34	85
6	682	Q8IVF4	Dynein axonemal heavy chain 10	DYH10_HUMAN	5.64	517677	14	57
7	828	Q6IBS0	Twinfilin-2	TWF2_HUMAN	6.3	39751	24	51
8	899	Q9NRC6	Spectrin beta chain, non-erythrocytic 5	SPTN5_HUMAN	6.2	419259	20	68
9	655	P05783	Keratin, type I cytoskeletal 18	K1C18_HUMAN	5.3	48029	69	184
10	896	P20674	Cytochrome c oxidase subunit 5A, mitochondrial	COX5A_HUMAN	6.3	16923	66	85
11	365	Q16891	MICOS complex subunit MIC60	MIC60_HUMAN	6.08	84025	46	165
12	428	A6NI56	Coiled-coil domain-containing protein 154	CC154_HUMAN	8.62	76422	26	66
13	606	Q15149	Plectin	PLEC1_HUMAN	5.74	533462	14	57
14	686	Q6ZNG1	Zinc finger protein 600	ZN600_HUMAN	9.4	85841	24	57
15	692	Q8NAA6	Putative uncharacterized protein encoded by LINC02694	CO053_HUMAN	9.9	20123	38	57
16	674	O75665	Centriole and centriolar satellite protein OFD1	OFD1_HUMAN	5.8	117055	12	58
17	696	Q96JY0	Protein maelstrom homolog	MAEL_HUMAN	8.9	49872	29	59
18	786	P35232	Prohibitin 1	PHB_HUMAN	5.5	29843	83	131
19	603	Q96JE9	Microtubule-associated protein 6	MAP6_HUMAN	9.2	86680	31	57
20	767	P21941	Cartilage matrix protein	MATN1_HUMAN	7.8	54465	27	58
21	581	P06576	ATP synthase subunit beta, mitochondrial	ATPB_HUMAN	5.2	56525	72	241
22	779	P13645	Keratin, type I cytoskeletal 10	K1C10_HUMAN	5.13	59020	29	63
23	465	P07437	Tubulin beta chain	TBB5_HUMAN	4.7	50095	60	185
24	605	Q9H2K2	Poly [ADP-ribose] polymerase tankyrase-2	TNKS2_HUMAN	6.6	128492	19	65
25	409	P35251	Replication factor C subunit 1	RFC1_HUMAN	9.3	128688	16	58

26	419	P10809	60 kDa heat shock protein, mitochondrial	CH60_HUMAN	5.7	61187	39	100
27	453	Q9Y2G4	Ankyrin repeat domain-containing protein 6	ANKR6_HUMAN	9.42	80763	20	58
28	695	Q9H8U3	AN1-type zinc finger protein 3	ZFAN3_HUMAN	7.4	26023	25	59
29	362	Q9H8U3	AN1-type zinc finger protein 3	ZFAN3_HUMAN	7.4	26023	38	73
30	637	P63261	Actin, cytoplasmic 2	ACTG_HUMAN	5.3	42108	74	178
31	791	P63104	14-3-3 protein zeta/delta	1433Z_HUMAN	4.7	27899	56	93
32	678	Q96HD9	N-acyl-aromatic-L-amino acid amidohydrolase (carboxylate-forming)	ACY3_HUMAN	5.5	35504	21	59
33	386	Q9NYC9	Dynein axonemal heavy chain 9	DYH9_HUMAN	5.6	515627	19	74
34	677	B1AJZ9	Forkhead-associated domain-containing protein 1	FHAD1_HUMAN	6.5	162659	20	72
35	847	Q7Z3Y8	Keratin, type I cytoskeletal 27	K1C27_HUMAN	5.06	50420	24	69
36	680	Q99959	Plakophilin-2	PKP2_HUMAN	9.3	97852	21	66
37	601	O95243	Methyl-CpG-binding domain protein 4	MBD4_HUMAN	9.01	66808	33	57
38	665	Q9UK32	Ribosomal protein S6 kinase alpha-6	KS6A6_HUMAN	5.9	84389	33	63
39	642	P43304	Glycerol-3-phosphate dehydrogenase, mitochondrial	GPDM_HUMAN	7.5	81315	25	57
40	814	P04792	Heat shock protein beta-1	HSPB1_HUMAN	5.9	22826	72	152
41	612	P63261	Actin, cytoplasmic 2	ACTG_HUMAN	5.3	42108	80	197
42	691	B2CW77	Killin	KILIN_HUMAN	11.2	20288	67	73
43	799	Q9NZQ3	NCK-interacting protein with SH3 domain	SPN90_HUMAN	5.9	79651	21	57
44	449	A2A3K4	Protein tyrosine phosphatase domain-containing protein 1	PTPC1_HUMAN	7.3	85543	27	57
45	707	Q8NBN7	Retinol dehydrogenase 13	RDH13_HUMAN	8.2	36195	33	63
46	414	O95613	Pericentrin	PCNT_HUMAN	5.39	380644	11	64
47	334	O95171	Sciellin	SCEL_HUMAN	9.42	78018	20	66
48	423	P07237	Protein disulfide-isomerase	PDIA1_HUMAN	4.7	57480	73	239
49	460	P12883	Myosin-7	MYH7_HUMAN	5.6	223757	24	57
50	804	Q9Y3D5	28S ribosomal protein S18c, mitochondrial	RT18C_HUMAN	9.6	16238	52	67
51	393	P25705	ATP synthase subunit alpha, mitochondrial	ATPA_HUMAN	9.16	59828	55	126
52	438	Q9H8U3	AN1-type zinc finger protein 3	ZFAN3_HUMAN	7.4	26023	28	59
53	912	O00571	ATP-dependent RNA helicase DDX3X	DDX3X_HUMAN	6.7	73597	29	59
54	547	P10809	60 kDa heat shock protein, mitochondrial	CH60_HUMAN	5.7	61187	38	70

55	790	Q8IY21	Probable ATP-dependent RNA helicase DDX60	DDX60_HUMAN	7.6	199680	14	57
56	792	Q9UKX3	Myosin-13	MYH13_HUMAN	5.5	224681	17	65
57	487	P10809	60 kDa heat shock protein, mitochondrial	CH60_HUMAN	5.7	61187	34	85
58	635	Q9Y623	Myosin-4	MYH4_HUMAN	5.6	223902	23	64

[a] Spot numbers
[b] Theoretical isoelectric point
[c] Theoretical relative mass
[d] MASCOT coverage
[e] MASCOT score
